# Increased Gamma Connectivity in the Human Prefrontal Cortex during the Bereitschaftspotential

**DOI:** 10.3389/fnhum.2017.00180

**Published:** 2017-05-02

**Authors:** Kisun Kim, June Sic Kim, Chun Kee Chung

**Affiliations:** Department of Brain and Cognitive Science, Seoul National UniversitySeoul, South Korea

**Keywords:** Bereitschaftspotential, readiness potential, electrocorticography (ECoG), connectivity, Partial directed coherence (PDC), prefrontal cortex, movement preparation, beta and gamma band

## Abstract

The Bereitschaftspotential (BP) is a slow negative cortical potential preceding voluntary movement. Since movement preparation is dependent upon the synchronous activity of a variety of neurons, BP may develop through the exchange of information among motor-related neurons. However, the relationship between BP and information flow is not yet well-known. In the present study, we aimed to investigate how the connectivity in the prefrontal cortex (PFC) changes during the occurrence of BP. Electrocorticography (ECoG) was recorded in five patients with epilepsy. The subjects performed self-paced hand grasping. We compared the intraregional connectivity between PFC and non-PFC regions using partial directed coherence. In the PFC, the connectivity of beta and gamma bands in the BP period increased by an average of 24.4% compared with the baseline connectivity. Conversely, gamma connectivity in non-PFC regions decreased by 31.4%. Moreover, the intraregional connectivity in the PFC increased according to the stage of BP. The increased gamma band connectivity in the PFC implies that the increased communication among neurons in the PFC is associated with development of BP. Intraregional connectivity as one of the factors involved in voluntary movement may reflect the activation of brain networks related to movement preparation in PFC.

## Introduction

Movement of the human body is more than simply muscle activity; it also involves higher cognitive function in the planning of when and how to move. For a precise voluntary movement, warm-up time is needed to gather information from environmental sensory signals and internal brain processes. In electroencephalography (EEG), this preparatory activity is observed as a slow and negative potential known as the Bereitshcaftspotential (BP) or the readiness potential (RP; Kornhuber and Deecke, 1965). BP reflects the intention, planning, and execution of a movement, and it begins up to 2 s prior to the onset of muscle movement (Shibasaki et al., 1980). BP has mostly been reported in the motor area (Deecke and Kornhuber, 1978; Roland et al., 1980; Neshige et al., 1988). However, according to more recent studies, the prefrontal cortex (PFC) is also an important area in the generation of BP (Rektor et al., 1994; Jahanshahi et al., 1995, 2001; Ryun et al., 2014).

Since BP may develop gradually through the accumulation of information from various brain areas, BP is not formed as a spike but as a gentle slope and is built up slowly compared with other brain responses such as evoked potentials. The more information a BP encodes about the intended movement, the larger the amplitude or latency (Morgan et al., 1992). Therefore, a BP associated with an accuracy movement may require sufficient time to receive information from the movement-related areas.

BP consists of two components with different slopes. One is the early BP that develops ~1,500 ms before the onset of a movement. Another is the late BP that follows the early BP, which develops ~500 ms prior to a movement and has a relatively steep slope (Neshige et al., 1988). While the early component was thought to be more specific for general preparation for movement, the late component was known to be related to the site of movement and its control (Shibasaki and Hallett, 2006). However, there are individual variants of these two BP components, depending on the movement because the late BP can be larger or smaller than the early BP. In addition, the onset latency of the early BP is reported to have a large inter-individual variability (Kukleta et al., 2012), implying that a movement may need different amounts of information, depending on the type of movement. Different information processes may result in the inter-individual and inter-conditional variability of the BP. Therefore, the BP may reflect the exchange of information and the change in connectivity among motor-related neurons for precise and intended movements.

Intraregional connectivity indicates a small network connectivity that shares similar work simultaneously, whereas interregional connectivity is long-range connectivity among different regions cooperating in identical goals, such as the sensory-motor network (Passingham, 1993; Dum and Strick, 2005) and the language network (Friederici, 2002, 2012). Participation of movement-related brain regions such as the PFC and the motor cortex is needed for planning a movement and for deciding the appropriate onset time. In addition, information flow within the movement-related regions may be involved in the process of diverse movements and would be reflected in the effective connectivity. This connectivity may underlie the development stage of BP for a specific movement. Thus, the intraregional effective connectivity within the movement-related region would be associated with BP generation.

The present study aimed to investigate the role of connectivity in the generation of BP in the PFC before movement. To do this, we have developed two hypotheses on the neural basis of BP. First, BP is generated by changes in intraregional effective connectivity in the PFC and an increase in BP is likely related to an increase in the amount and strength of these connectivities. Second, there are comparable connectivity differences between the PFC and the non-PFC areas during BP.

## Materials and methods

### Subjects

Prior to the study, all subjects submitted written consent for participating in the study. This study was approved by the Institutional Review Board of the Seoul National University Hospital (IRB No. H-0912-067-034). Five subjects (three females and two males, aged 25–37 years) with intractable epilepsy participated in the present study. All patients underwent implantation of subdural electrodes for clinical purposes. The clinical demography of the patients is presented in Table 1. Computed tomography (CT, Siemens SOMATOM sensation16, Siemens medical solution, Erlangen, Germany) for each subject was carried out before and after implantation of the subdural electrodes. Magnetic resonance images (MRIs, GE Signa 3T scanner, GE medical system, Milwaukee, Wisconsin) were also acquired after the implantation.

**Table 1:** **Clinical profiles**.

**Subject**	**Side of hand movement**	**Location of intracranial electrodes**	
		**Location**	**Number**
1	Left	Right F,P,O	88
2	Left	Right F,P	52
3	Right	Left F,T	48
4	Left	Right F,P,O,T	82
5	Left	Right F,P,T	58

*F, frontal; P, parietal; T, temporal; O, occipital*.

### Experimental protocols and data acquisition

The subjects were instructed to perform self-paced, contralateral hand grasping to the implantation hemisphere. They performed hand grasping in accordance with instructions that required the collection of sufficient inter-trial intervals that were >5 s. The patients were instructed not to count the number of seconds in an interval, and we emphasized the importance of movement intention immediately before performing the movement. The patients performed hand grasp and release 25–30 times during one session. Three sessions were recorded for each patient, with each session taking ~5 min, followed by a 2 min rest between sessions.

Each patient had 48–88 subdural electrodes (Ad-tech Medical Instrument Co., Racine, WI, U.S.A), with each electrode having a diameter of 4 mm and an inter-electrode distance of 10 mm. The brain model and implanted electrodes were reconstructed from the individual MRI and CT images using CURRY (version 8.0, Compumedics Neuroscan, U.S.A.). The Electrocorticography (ECoG) was recorded using a digital video EEG monitoring system (Telefactor Beehive Horizon with an AURA® LTM 64 and 128-channel amplifier system, West Warwick, Rhode Island, U.S.A.), digitized at sampling rates of 400 or 1,600 Hz and filtered from 0.1–100 Hz. The cheek bone was used as a reference. Additionally, electromyography (EMG) was carried out to detect the onset of the movement of the *opponens pollicis* during hand grasping. Electrooculography (EOG) was also carried out to monitor simultaneously eye-blinking and eye-movement. The entire experiment was video-recorded to monitor motor performance and to establish a more precise definition of movement onset (Ryun et al., 2014). The ECoG channels showing abnormal signals caused by pathology or technical problems were excluded from further analyses.

### Signal preprocessing

**Figure 1:**
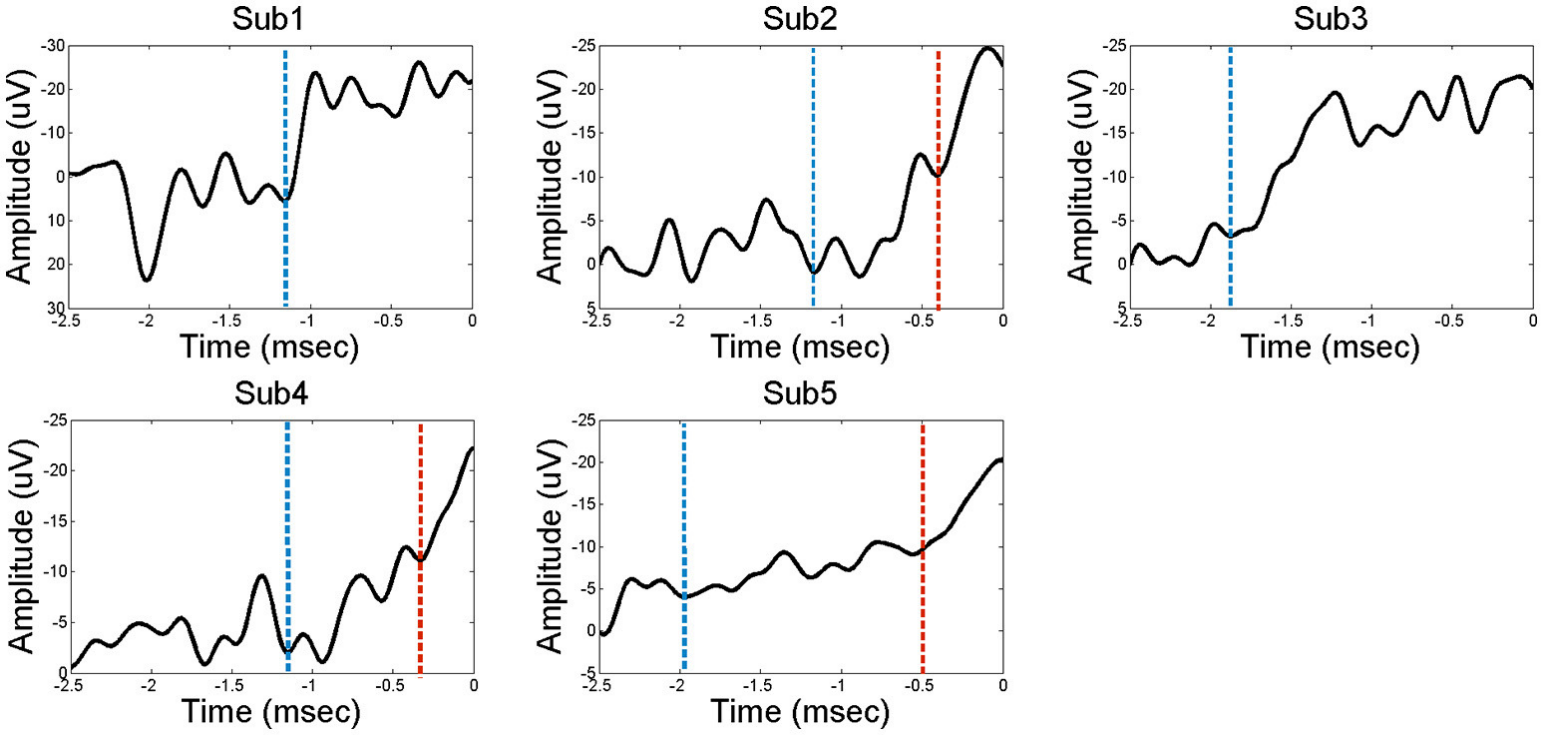
**The Bereitshcaftspotential (BP) of five subjects**. Each of the electrodes in the PFC represents a feature of BP; a classical two components before movement. Dashed line in blue indicates the starting point of early BP. Dashed line in red indicates the starting point of late BP. Subjects 2, 4, and 5 have distinct early BP and late BP stages, while subjects 1 and 3 have undistinguishable BP component.

The ECoG data were analyzed using Matlab software (Mathworks, Natick, MA). The recorded data were down-sampled to 200 Hz for unification of the various sampling rates in the analysis. After the recording, we re-referenced the signals using a common average reference (CAR) to reduce artifacts that globally influenced ECoG electrodes. The movement onset was determined based on the EMG signals. All of the trials were averaged with a time window of 5 s covering a period from 4 s before movement onset to 1 s after. Finally, channels that represented the features of BP were chosen among the electrodes in the PFC. Figure 1 demonstrates representative examples of “classical” two-component intracerebral BPs. The waveform in Subject 5 has the following typical BP characteristics: (1) The early BP shows an initial slowly increasing negativity with a minimally discernible onset. (2) The late BP becomes steeper than the early BP and appears ~500 ms before the task-relevant EMG activity. For the connectivity analysis, the time windows of 500 ms were separately selected in the early BP, late BP, and baseline period. Each time window was selected in the middle of each component. To compare the intraregional connectivities between the PFC and the non-PFC regions, non-PFC electrodes were selected outside the PFC and the motor related area. Non-PFC electrodes in each subject were located as follows; subject 1: parieto-occipital lobe, subject 2: parietal lobe, subject 3: temporal lobe, subject 4: parieto-occipital lobe, subject 5: parietal lobe.

### Connectivity analysis

Partial directed coherence (PDC) was used for the connectivity analysis (Baccala and Sameshima, 2001). PDC is a connectivity estimator in the frequency domain that is based on a multivariate autoregressive (MVAR) model. This model provides a linear measure of causality representing the direction and strength of the interaction among multiple variables. Under self-paced movement conditions, averaged data across all trials were used for the connectivity analysis. PDC estimates the connectivity patterns among subdural electrodes. According to the MVAR model, a multivariate process can be described as a data vector, *S*, of *M* source signals in time, *t*:

$$\begin{array}{l} S\left(t\right)={\left(S_{1}\left(t\right),S_{2}\left(t\right),\cdots \;,S_{M}\left(t\right)\right)}^{T}. \end{array}$$

The MVAR model can then be constructed as:

$$\begin{array}{l} S\left(t\right)=\overset{m}{\underset{n=1}{\sum }}P\left(n\right)S\left(t-n\right)+E\left(t\right) \end{array}$$

where *E*(*t*), the vector of white noise at time *t*, denotes the error of the MVAR model. *P*(*n*) is an *M* × *M* matrix that includes the model coefficients, and *m* is the model order.

The MVAR model was transformed into the frequency domain as follows:

$$\begin{array}{l} P\left(f\right)S\left(f\right)=E\left(f\right) \end{array}$$

where *f* denotes frequency and $P\left(f\right)=-\sum_{n=0}^{m}P\left(n\right)e^{-i2\pi fn\Delta t}$ with *P*(0) = *I* (*I* is the identity matrix). The PDC factor from *j* to *i* is given by

$$\begin{array}{l} \text{PDC }\left(f\right)=\frac{P_{ij}\left(f\right)}{\sqrt{p_{j}^{\text{H}}\left(f\right)p_{j}\left(f\right)}} \end{array}$$

where *P*_*ij*_(*f*) is the *i, j-*th element of *P*(*f*), *P*_*j*_(*f*) is the *j*-th column vector, and $P_{j}^{\text{H}}\left(f\right)$ is the conjugate transpose of *P*_*j*_(*f*).

PDC-values are represented as a range from 0 to 1. Values close to 1 indicate that most of the signal in source *i* is caused by the signal from source *j*. Conversely, values close to 0 indicate that there is little information flow from source *j* to *i* at a particular frequency, *f*. The distribution of PDC estimators is not well-established because PDC functions have a highly nonlinear relationship with time series data. Therefore, we employed a non-parametric statistical estimation method. A surrogate data technique was used to test the significance of propagation among activities. The surrogate technique randomly and independently shuffles the time series data from each source to create a surrogate data set. To shuffle time series data, we randomized phases in the Fourier transformed signal to leave the power spectra of source signals intact. Propagation was then derived from this surrogate data set. By performing this process 10,000 times, we were able to create an empirical distribution for a given estimator. These distributions provided null hypotheses, since the randomly generated surrogate data set contained no interaction among sources. In the present study, mean values of multiple frequencies and individual frequencies from 1 to 50 Hz were used to reconstruct empirical distributions. Detailed information regarding the method is described in our previous study (Kim et al., 2010). We used a window size of 500 ms to estimate the PDC. PDCs of different time ranges (e.g., baseline, early BP, and late BP) were estimated by sliding the window. If the onset of late BP was barely distinguishable (e.g., subject 1 and 3 in Figure 1), the window of 500 ms before the movement onset was used on analysis as the late BP. The PDC spectra were analyzed from 1 to 50 Hz, which were further divided into sub-bands—delta (1~3 Hz), theta (4~7 Hz), alpha (8~12 Hz), beta (13~30 Hz), and gamma (31~50 Hz). High gamma (>50 Hz) may be useful to decode movement type and better reflects functional localization of movement. However, the cut-off frequency of the anti-aliasing analog filter was 100 Hz in this study. Therefore, it was difficult to include high gamma frequency in the analysis. We plan to include high gamma frequency for further studies. The statistical significance of the PDC-value was determined by fisher's *z*-transformation at a significance level of 0.05. The differences between the baseline and BP stage were tested statistically using the Wilcoxon signed rank test for non-parametric testing.

## Results

### The connectivity change according to the BP stage in the PFC

**Figure 2:**
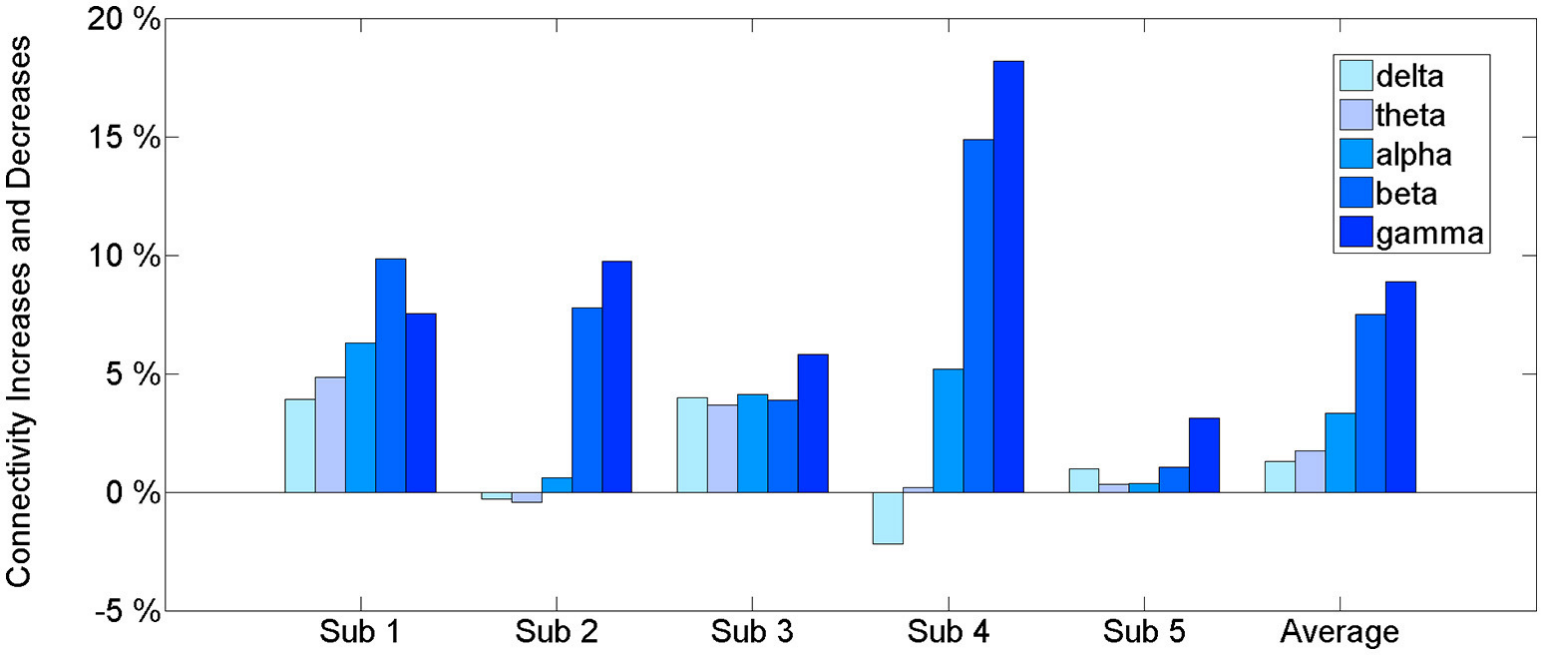
**The connectivity change of frequency bands during a late BP in the PFC**. An estimated value of PDC is between 0 and 1. The information that we want to give is the connectivity increase in BP period compared to baseline. Therefore, we suggest percentage change for relative comparison not only just original value. See Supplementary Data for original value.

The results of the connectivity regarding the frequency bands show a difference between the baseline and BP phases in the ECoG records of five patients. Table 2 and Figure 2 show the percentage increase in connectivity in the PFC according to each frequency band. Compared to the baseline, most of the frequency bands show increasing connectivity during the BP. In particular, remarkable increases were present in the beta and gamma band during late BP.

**Table 2:** **Connectivity change of frequency bands during a late BP in the PFC**.

		**Delta**	**Theta**	**Alpha**	**Beta**	**Gamma**
Sub1	Value (%)	0.018	0.023	0.03	0.047	0.036
		7.8	9.71	12.56	19.72	15.06
Sub2	Value (%)	0.001	0.002	0.002	0.027	0.033
		−0.57	−0.85	1.24	15.55	19.51
Sub3	Value (%)	0.019	0.019	0.022	0.022	0.031
		7.96	7.36	8.27	7.77	11.62
Sub4	Value (%)	0.01	0.001	0.024	0.064	0.066
		−4.37	0.38	10.35	29.76	36.36
Sub5	Value (%)	0.006	0.008	0.016	0.032	0.036
		2.56	3.45	6.63	14.98	17.76

### Quantification of gamma connectivity in late BP: comparison between PFC and non-PFC

**Figure 3:**
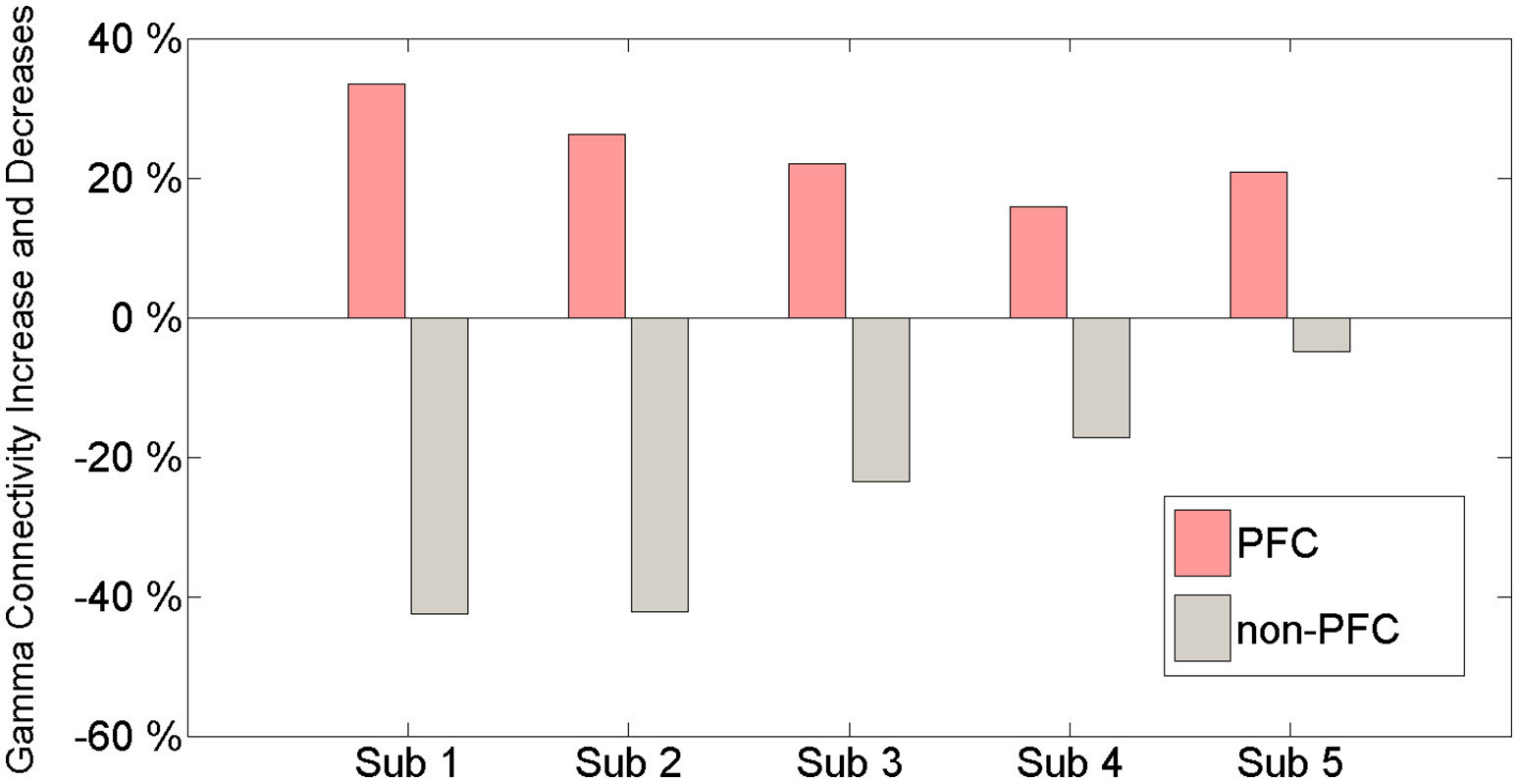
**The amount of gamma connectivity increases and decreases in comparison with the baseline and BP between the PFC and the non-PFC regions**.

Gamma band connectivity in the PFC of five patients revealed a marked increase compared with that in the non-PFC regions. In comparison with the baseline, during the build-up to a BP average gamma connectivity increased by 24.4% in the PFC, whereas it decreased by 31.4% in the non-PFC regions. Subject 1 showed a 33.5% increase in the PFC and a 42.4% decrease in the non-PFC regions. Subject 2 showed a 26.3% increase in the PFC and a 42.2% decrease in the non-PFC regions. Subject 3 showed a 22% increase in the PFC and a 24% decrease in the non-PFC regions. Subject 4 showed a 15.9% increase in the PFC and a 17.2% decrease in the non-PFC regions. Subject 5 showed a 20.8% increase in the PFC and a 4.9% decrease in the non-PFC region. Figure 3 shows that, compared to the baseline, gamma connectivity increases and decreases for BP in the PFC and non-PFC regions.

### Spatial patterns of gamma connectivity in the PFC and non-PFC regions

**Figure 4:**
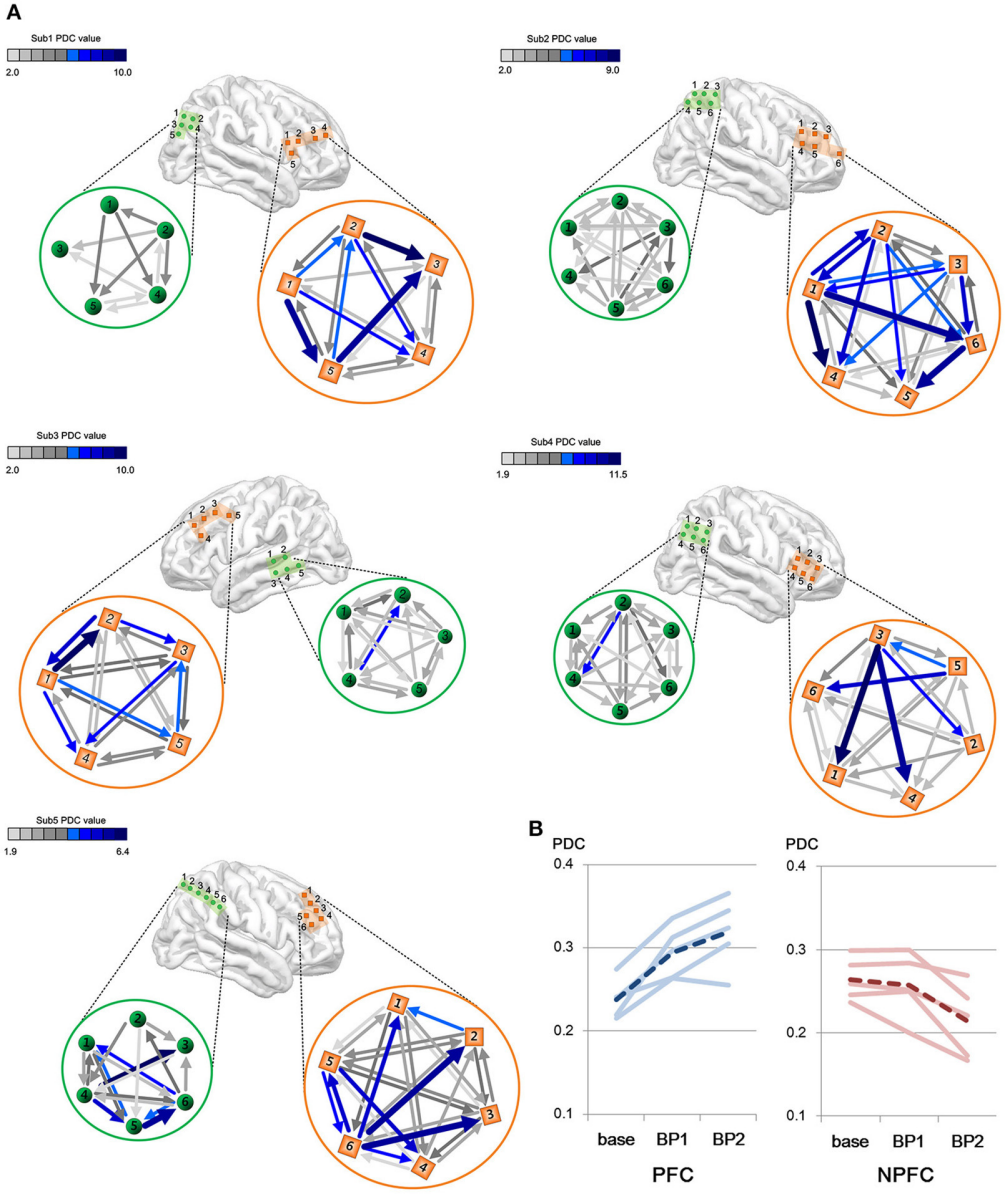
**(A)** Differences in gamma connectivity strength between the PFC and the non-PFC regions during the BP stages. Each of brain maps represents ECoG position of each subject. Color bar shows the strength of connectivity from the PDC. Light green color indicates a weak PDC connectivity, whereas a dark blue color indicates a strong PDC connectivity. Each connectivity value is calculated by PDC measures and a PDC value of *P* < 0.05 is considered as significant and represented as arrows in the figure. **(B)** Change in gamma connectivity by the BP development stage. Blue lines indicate PDC value in the PFC. Red lines indicate PDC value in the non-PFC regions. Solid lines indicate individual subjects' connectivity change. Dashed lines indicate the mean of five subjects' PDC change.

We consider a PDC-value of *p* < 0.05 to be significant by Fisher's *z*-transformation. We compared the intraregional connectivity in the PFC to that in the non-PFC regions. Figure 4A shows the difference in amount and strength of connectivities between the PFC and the non-PFC regions during the BP stage. All subjects show a much stronger connectivity in the PFC area than in the non-PFC areas.

The amount of connectivity of intraregional BP increased in proportion to the BP stage (Figure 4B). In the case of the PFC, the mean PDC-values are 0.238 in the baseline, 0.295 in the early stage of BP and 0.319 in the late stage of BP. In the case of the non-PFC, the mean PDC-values are 0.264 in the baseline, 0.257 in the early stage of BP, and 0.214 in the late stage of BP. Note that gamma connectivity in the PFC increased until immediately prior to movement onset (*p* < 0.05, Wilcoxon signed rank tests), whereas gamma connectivity in the non-PFC decreased (*p* < 0.05, Wilcoxon signed rank test).

## Discussion

### Relationship between BP and connectivity

The intraregional connectivity is involved in building BP. This study shows an increased beta and gamma connectivity among multiple ECoG channels in the human PFC immediately before the self-paced movement. The increased connectivity indicates the strong neuronal communication within the PFC that occurs during movement preparation. The information exchange at the neuronal level induces synchronous cortical activity. Thus, the intraregional connectivity would be accompanied by BP. Our first analysis (Figure 2) of an increased PFC network connectivity during self-paced movement supports the idea that connectivity is associated with the generation of BP. In addition, this increase in connectivity occurred dominantly in the gamma band (30–50 Hz), suggesting that the gamma band initiates the interactions in the PFC.

The connectivity in the BP period is dynamic: the more the connectivity increases, the bigger is the BP, as shown in Figure 4B. In other words, connectivity increases over time prior to movement onset. These simultaneous increases in the BP and intraregional connectivity occurred consistently in all five subjects, implying that the neuronal activity is synchronized according to continuous and accumulating information flow. Wu et al., showed that a Parkinson's disease (PD) patient who had a problem with movement execution had less connectivity among motor-related areas, including the PFC (Wu et al., 2011), which indirectly demonstrates that the interaction of brain networks is disrupted in PD patients during the performance of self-paced movement. However, Wu et al.'s (2011) study did not present the relationship between BP and connectivity. Our report is the first to show the relationship between intraregional connectivity and BP.

### The role of PFC for BP

It is generally accepted that the PFC plays a role in the preparation of voluntary movement (Passingham, 1996; Jueptner et al., 1997). Increased movement intention is associated with strong activation of the pre-SMA region (Lau et al., 2004). Conversely, a lesion in the PFC area including parts of the DLPFC can lead to a decline or a complete absence of BP preceding movement (Singh and Knight, 1990; Wiese et al., 2004), indicating the loss of attention and motivation associated with abnormality in these regions (Singh and Knight, 1990; Wiese et al., 2004). In our present study we found a BP only in the PFC region, as shown in Figure 1. Moreover, intraregional connectivity was correlated with BP in the PFC, but not in the non-PFC regions. Conversely, the connectivity during BP period was decreased in the non-PFC regions, implying that the PFC is a key region in the generation and build-up of the BP by means of reinforcement of information exchange.

### The role of gamma activity in BP

The present results demonstrate significant beta and gamma band connectivity in the PFC. The most prominent connectivity increase during development of the BP was found in the gamma band. Extensive studies support the fact that gamma oscillation is related to motor and sensory processing required for cognitive execution of the task. Gamma oscillations have been described associated with a variety of sensory functions (Cardin et al., 2009), including perceptual learning (Gruber et al., 2002) and movement execution (Cheyne et al., 2008). Recent studies have reported that gamma oscillation is elicited during voluntary movement in primary motor cortex (Cheyne et al., 2008) and anterior supplementary motor area (Ball et al., 2008). Gamma oscillation induced by voluntary movement indicates activation of cortico-subcortical networks involved in the feedback control of movements (Cheyne et al., 2008). In addition, an increase in cortico-muscular coherence in the gamma band was observed in the motor cortex during preparation of the response to a visual stimulus (Schoffelen et al., 2005). In short, gamma oscillation may reflect various temporal dynamics of cortical networks and their interactions. Gamma oscillation activity has been found over the frontal lobe during a top-down task. Gamma may reflect the activation of executive networks involved in decision making (Kaiser and Litzenberger, 2004). With regard to connectivity, slow oscillations such as alpha, delta, or theta have been observed in long-range connectivity, whereas fast oscillations such as gamma are involved in short-range connectivity. Mostly, gamma connectivity takes part in local recruitment and task-related functions (von Stein and Sarnthein, 2000; Buzsaki and Draguhn, 2004). When considering the role of gamma oscillation, gamma connectivity, which is increased with the development of a BP, may reflect information flow for the preparation of movement. Beta band also contributes to the generation of BP; it is the second most associated signal, after the gamma band. Self-paced voluntary movement is preceded by decreases in the power of EEG activities in the beta frequency, which is known as event-related desynchronization (ERD; Doyle et al., 2005).

### Development of early and late BPs

BP is divided into early and late stages. During the progression from early to late BPs, the strength of intraregional connectivity increases (i.e., the connectivity in the late BP is stronger than that in the early BP, Figure 4), implying that BP and connectivity are closely related. In the early BP, decisions regarding the intention and timing were made mainly in the PFC. Thus, decision information may accumulate and elevate the intraregional connectivity in the PFC. The late BP, which is thought to be more specific to aspects related to preparation of movement, including precision, discreteness, and complexity, requires more information for complex movement execution and increases more steeply than the early stage. Consequently, our results suggest that fine movement may finally be prepared through information exchange.

### Connectivity decreases in the non-PFC regions during BP

In the connectivity results, we found that connectivity increased simultaneously with BP in the PFC area. In contrast, the connectivity decreased in the non-PFC regions (Figure 3). This result may be interpreted as an aspect of distribution of attention. The connectivity in the non-PFC regions that are not involved in BP generation was decreased by attention during movement preparation. Jiang et al., showed that the connectivity of movement-related areas such as the left M1, left PMC, and left SPL increased, but the connectivity of the left superior cerebellum, left dentate nucleus, right cuneus, and left basal ganglia decreased during the movement state compared to the resting stage (Jiang et al., 2004).

### Limitations

There are three concerns that should be discussed in interpretation of our results. Firstly, in the PDC analysis, the meaning of connectivity at a frequency band is not directly related to the activity at the specific frequency band. Connectivity using an MVAR model is more sensitive to the change of signal phase than amplitude. Our results from the PDC analysis do not represent levels of gamma oscillation activity itself but connectivity in the gamma band. Nevertheless, we showed an increase of the intraregional connectivity in the PFC in proportion to the BP stages. Further, study may be needed to investigate the correlation between connectivity and BP.

The second issue is that the increase of connectivity may be affected by the volume conduction effect. Similar signals can be picked up at adjacent electrodes, causing an increase of connectivity. However, we compared the connectivity between different time windows and different regions at the same electrode distribution. For the temporal difference, we compared the connectivity among the baseline, the early BP, and the late BP. For the spatial difference, the connectivity within the PFC area was compared with the connectivity within the non-PFC area. Thus, the change in connectivity in our study is not likely caused by the volume conduction influence.

Lastly, our study suggests that the intraregional connectivity may be one of factors that contribute to building the BP. The mechanism of BP generation is not well-known. The sources of BP include primary motor cortex, supplementary motor area, and PFC. We attempted to demonstrate the relationship among motor-related regions, to suggest a possible mechanism of BP generation. However, this approach is insufficient to prove the mechanism because the study does not establish causation. The time domain BP might be a different phenomenon from the frequency domain connectivity studied here. In a further study, we expect to perform modeling or simulation studies to reinforce the hypothesis.

## Conclusions

In conclusion, this is the first study that demonstrates increased gamma connectivity among multiple ECoG channels in the human PFC during self-paced movement. The PFC has an effect on the modulation of movement—an aspect of movement preparation that involves a progressive build-up in gamma oscillation connectivity. Gamma oscillation plays a role in binding multiple inputs from a diverse area (Engel and Singer, 2001). Our results demonstrate that intraregional connectivity is involved in the build-up of a BP and suggest that the amount of information in the PFC is important for the preparation of movement.

## Author contributions

All authors participated in experiments, analysis, and writing, and approved the final version to be published. JK had full access to all of the data in the study and takes responsibility for the integrity of the data and accuracy of the data analysis. Study conception and design: KK, JK, CC. Acquisition of data: KK, JK, CC. Analysis and interpretation of data: KK, JK, CC. Manuscript preparation: KK, JK.

### Conflict of interest statement

The authors declare that the research was conducted in the absence of any commercial or financial relationships that could be construed as a potential conflict of interest.
