# Supplementary material for: Increased Gamma Connectivity in the Human Prefrontal Cortex during the Bereitschaftspotential
Source: Front Hum Neurosci. 2017 May 2;11:180. doi: 10.3389/fnhum.2017.00180 (PMC5411441; doi:10.3389/fnhum.2017.00180)
Supplement: Supplementary file 1 [file Table1.DOCX]

**Supplementary data**

|  | **Delta** | | **Theta** | | **Alpha** | | **Beta** | | **Gamma** | |
| --- | --- | --- | --- | --- | --- | --- | --- | --- | --- | --- |
|  | Baseline | BP | Baseline | BP | Baseline | BP | Baseline | BP | Baseline | BP |
| **Sub1** | 0.2297 | 0.2476 | 0.2357 | 0.2586 | 0.2409 | 0.2712 | 0.2398 | 0.2781 | 0.2421 | 0.2785 |
| **Sub2** | 0.1882 | 0.1872 | 0.1925 | 0.1909 | 0.1926 | 0.1950 | 0.1752 | 0.2025 | 0.1716 | 0.2052 |
| **Sub3** | 0.2427 | 0.2620 | 0.2547 | 0.2734 | 0.2654 | 0.2874 | 0.2797 | 0.3014 | 0.2658 | 0.2967 |
| **Sub4** | 0.2322 | 0.2220 | 0.2339 | 0.2348 | 0.2288 | 0.2525 | 0.2029 | 0.2633 | 0.1824 | 0.2487 |
| **Sub5** | 0.1958 | 0.1997 | 0.2106 | 0.2120 | 0.2259 | 0.2275 | 0.2259 | 0.2306 | 0.2123 | 0.2256 |

Results of PDC connectivity of frequency bands show a difference between the baseline and BP phases in the ECoG records of 5 patients. An estimated value of PDC is between 0 and 1. The information that we want to give is the connectivity increase in BP period compared to baseline. Therefore, we suggest percentage change for relative comparison not only just original value in manuscript. Original values are written below as a table.
